# The microstructure matters: breaking down the barriers with single crystalline silicon as negative electrode in Li-ion batteries

**DOI:** 10.1038/srep31712

**Published:** 2016-08-17

**Authors:** M. Sternad, M. Forster, M. Wilkening

**Affiliations:** 1 Christian Doppler Laboratory for Lithium Batteries, Institute for Chemistry and Technology of Materials, Graz University of Technology (NAWI Graz), Stremayrgasse 9, 8010 Graz, Austria; 2 Infineon Technologies Austria AG, 9500 Villach, Austria; 3 ALISTORE-ERI European Research Institute, 33 rue Saint Leu, 80039 Amiens, France

## Abstract

Silicon-based microelectronics forms a major foundation of our modern society. Small lithium-ion batteries act as the key enablers of its success and have revolutionised portable electronics used in our all everyday’s life. While large-scale LIBs are expected to help establish electric vehicles, on the other end of device size chip-integrated Si-based μ-batteries may revolutionise microelectronics once more. In general, Si is regarded as one of the white hopes since it offers energy densities being ten times higher than conventional anode materials. The use of monocrystalline, wafer-grade Si, however, requires several hurdles to be overcome since it its volume largely expands during lithiation. Here, we will show how 3D patterned Si wafers, prepared by the sophisticated techniques from semiconductor industry, are to be electrochemically activated to overcome these limitations and to leverage their full potential being reflected in stable charge capacities (>1000 mAhg^–1^) and high Coulomb efficiencies (98.8%).

In the chase for higher energy densities the specific capacity of the anode material in lithium-ion batteries (LIBs) plays a major role. While graphite with its specific charge density of 372 mAhg^−1^, referring to the formation of LiC_6_^1^, represents the today’s state-of-the art anode material of most of the commercially available LIBs, the capability of silicon to take up Li ions is by far much higher. Charging a lithium-ion battery full cell with Si as the negative electrode lead to the formation of metastable^2^ Li_15_Si_4_; the specific charge density of crystalline Li_15_Si_4_ is 3579 mAhg^−1 ^3, which is almost ten times higher than that of graphite. Initially, entirely amorphous Li_*x*_Si is formed during the first lithiation step; subsequently, if the potential is kept below 50 mV *vs.* Li/Li^+^, the Li_15_Si_4_ phase shows up during the second step^3^,^4^.

The main issue that inhibits so far the real-life application of silicon as practicable anode in LIBs is the remarkable, to some extent inhomogeneous, increase in volume during lithiation. This increase amounts up to 300% if we refer to amorphous silicon (*a*-Si) and complete electrochemical lithiation (0 mV vs Li/Li^+^)^5^. Both internal mechanical stress and the dramatic expansion leads to structural damage especially after several charge-discharge cycles have been completed. To deal with this issue and to diminish dilatation one may take advantage of thin Si films^6^,^7^,^8^ and nanostructured or porous materials with their ‘free’ volume, *i.e.*, to make use of small, nm-sized Si particles^9^,^10^,^11^,^12^. Moreover, it is favorably to enduringly keep Si in the amorphous (*a*-Si) rather than in the crystalline state (*c*-Si) while the battery is subject to charging and discharging^5^,^13^,^14^. The use of nanostructured Si buffers not only volume expansion but also helps shorten diffusion paths for both Li ions and electrons. The large surface area of active material, however, may result in unwanted side reactions and heavy formation of passivating interphases. These so-called solid-electrolyte interphases (SEI) form because of the electrochemical instability of bare Si surfaces being in contact with the commonly used liquid electrolytes.

In contrast to these approaches, the present study proposes the direct use of single crystalline acceptor-doped Si as it is ubiquitously used in semiconductor industry. In our opinion, the use of patterned monocrystalline Si (*m*-Si) anodes, being directly shaped out of the Si wafer by means of the sophisticated manufacturing techniques of semiconductor industry, is a highly attractive route to realise miniaturised, on-board, *i.e.*, fully integrated, power supplies for Si-based chips. Against possible objectives regarding the electrochemical activity of monocrystalline Si, we will show how single crystalline Si in a well-defined microstructured form can serve as powerful, long-lasting Si electrode that does not need any binders or conductive additives. For this purpose, μm-sized towers, with dimensions being larger than 10 μm, were fabricated and their electrochemical performance tested. Although the monocrystalline towers cannot benefit from nanosize effects, several outstanding properties make them superior to nanostructured Si especially if microbatteries are considered.

As has been shown recently via *in situ* atomic scale imaging, during the first lithiation process *m*-Si transforms to an *a*-Li_*x*_Si-phase via a so-called “ledge-mechanism”, *i.e.*, by peeling off Si layer after Si layer^15^. Fortunately, Li ion diffusivity in *a*-Li_*x*_Si is rather high making it a convenient active material^16^. Subsequent delithiation of *a*-Li_*x*_Si yields the desired amorphous form of Si being characterised by the advantages sketched above^17^. Hence, if we could make use of *a*-Si electrochemically obtained from wafer-grade Si directly, the well-established manufacturing methods of semiconductor industry can be utilised to pre-fabricate structured Si anodes with crystal orientations perfectly supporting the preferred lithiation pathway. Such implemented batteries may be produced in quantities of up to 5000 cells per 8-inch wafer, *i.e.*, in a massively-parallel way. The low effort would result in low unit costs.

## Results and Discussion

### Patterned electrodes of single crystalline Si

**Figure 1:**
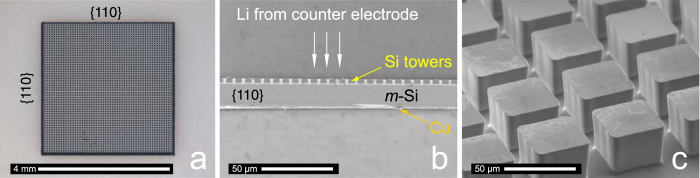
The m-Si electrode. **(a)** top view (4 × 4 mm^2^), **(b)** cross section and **(c)** the 3D patterned surface in detail.

The sophisticated production technology of Si-based semiconductor industry offers several routes to design the Si anode material. Using deep reactive-ion etching a boron-doped 8-inch silicon-wafer was surface-structured to yield towers with a quadratic base area of 50 × 50 μm^2^ and a height of 32 μm; the distance between the towers was 17.5 μm (see Fig. 1). To provide best possible electric contact to the Swagelok cell’s current collector (see below), the backside of the silicon wafer was sputter-coated with a 1 μm thick copper layer. Sawing the wafer to quadratic samples with a dimension of 4 × 4 mm^2^ completed the production process (Fig. 1).

### Electrochemical performance

**Figure 2:**
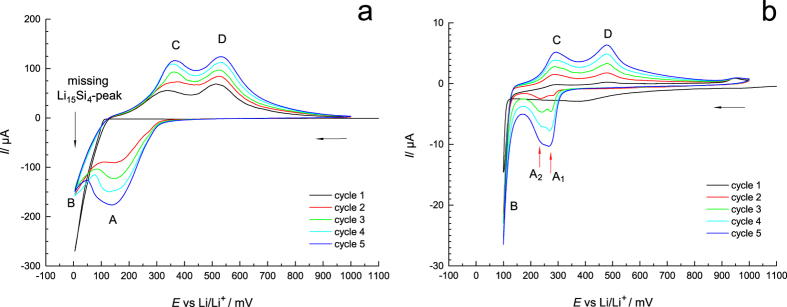
Cyclic voltammograms (5 cycles) of monocrystalline Si vs Li/Li^+^. **(a)** Voltammograms covering potentials *E* ranging from 1000 mV to 5 mV and **(b)** from 1000 mV to 100 mV. The scan rate was 10 μVs^–1^ (Mos1940, 1936).

Figure 2 shows the cyclic voltammograms (CVs) obtained for monocrystalline silicon for the 5 cycles carried out; the potential was varied between 1 V and 5 mV. Worth noting, Prior to the electrochemical tests the Si side of the electrodes was treated with hydrofluoric acid to remove any SiO_2_ layers^18^. During the initial reduction (black curve, see negative currents) a remarkable overvoltage is visible manifesting in lithiation currents showing up at potentials as high as 100 mV vs Li/Li^+^. Most likely, this behaviour is caused by the initially closed surface of the diamond-like crystal lattice that breaks up during the first lithiation forming amorphous regions. Subsequently, with increasing cycle number the electrode gains in electrochemical activity.

After the first cycle the lithiation process occurs in two steps, *cf*. peaks A and B showing up at 140 mV and 5 mV, respectively); both represent the transformation of Si into amorphous Li_*x*_Si. Although we recorded the CVs at a very low scan rate of 10 μVs^–1^ no discrete peak that could be ascribed to the formation of Li_15_Si_4_ was detected around 5 mV. This observation is in contrast to that of Baggetto *et al*. who studied polycrystalline thin films of Si^17^. Our finding that no extended regions of crystalline Li_15_Si_4_ were formed could be corroborated by *ex situ* X-ray diffraction, the corresponding pattern does not provide any evidence for the formation of crystalline Li_15_Si_4_. Compared to Baggetto *et al*.^17^ we deal with much thicker electrodes whose surface regions were primarily lithiated. The inner, crystalline regions remain untouched and act as a current collector. As can be seen at higher potentials, de-lithiation took place according to a two-step process, see peaks C and D showing up at ca. 360 mV 530 mV, respectively (Fig. 2a). If cyclic voltammetry is carried out between 1000 mV and 100 mV (see Fig. 2b) much lower currents are obtained (see Fig. 2a for comparison); the corresponding electrochemical activity turned out to be only slightly enhanced. However, the significant higher resolution uncovers that the first reduction peak A (see Fig. 2a) actually consists of two peaks, marked in Fig. 2b as A_1_ (270 mV) and A_2_ (240 mV). The appearance of at least three discrete reduction peaks (A_1_, A_2_, and B) in X-ray amorphous Li_*x*_Si points to the formation of distinct short-range ordered Li-rich clusters embedded in the *a*-Li_*x*_Si matrix. This excellently agrees with earlier results reported by Limthongkul *et al*.^19^ who used high-resolution transmission electron microscopy (TEM) to discover such clusters with diameters of only a few nm.

**Figure 3:**
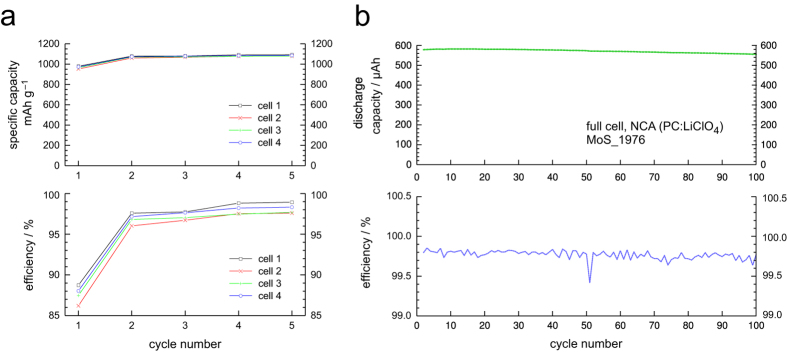
Charge-/discharge capacities and Coulomb efficiencies of the monocrystalline Si towers. Capacities larger than 1000 mAhg^−1^ and efficiencies as high as 98% were reached in half-cell configuration **(a)**. The solid lines are drawn to guide the eyes. In **(b)** long-term cycling is shown for a full-cell with NCA as cathode material. In full-cell configuration the absolute capacity (>500 μAh) is simply limited by the mass of the NCA used. Increasing its amount would result in higher discharge capacities.

In Fig. 3 the results of the cycling experiment are presented. Since lithium is stored in the tower-shaped parts of the electrode (see below and Experimental), specific discharge capacities as high as 980 mAhg^–1^ could be reached during the 1^st^ cycle; the Coulomb efficiency was 89%. After the 1^st^ cycle the capacity slightly increases reaching 1093 mAhg^–1^ (5^th^ cycle, 98.9% Coulomb efficiency); for the calculation of specific capacities see section Methods and Experimental. Worth mentioning, we tested the patterned anode also in full cell configuration using Li(Ni_0.80_Co_0.15_Al_0.05_)O_2_ (NCA) as cathode; as for the half cells a 1M solution of LiClO_4_ in propylene carbonate served as electrolyte (see Experimental). The full cell can be charged and discharged in a stable way for more than 100 times with only a marginal capacity fade. The Coulomb efficiencies are as high as 99.7%.

Most likely, the excellent Coulomb efficiency of already 89% during the 1^st^ cycle of the half cells (see Fig. 3a) is due to the low specific surface area of the anode material. Because no grain structure is present the surface exposed to the electrolyte is small at the beginning. Thus, volume expansion has only minor effects on the charge/discharge efficiency.

**Figure 4:**
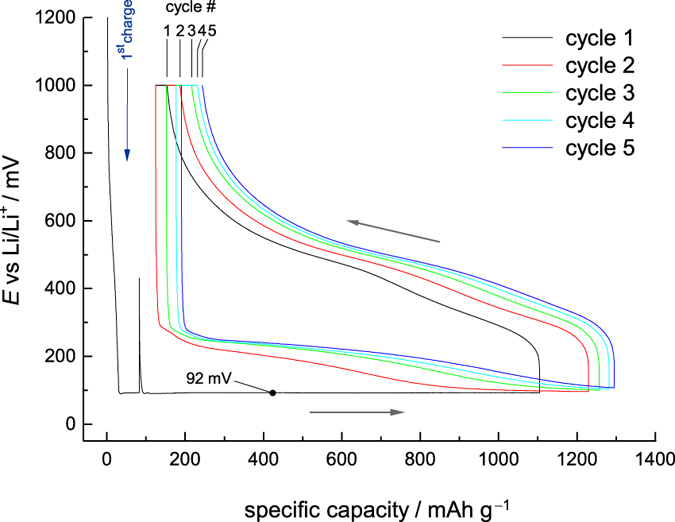
Charge/discharge curves. Voltage *vs.* specific capacity of monocrystalline silicon. *E* refers to metallic Li (Mos_1852).

Most importantly, at the beginning, the closed surface of the m-Si anode experiences a so-called *activation process* with regard to electrochemical performance. This activation process takes place primarily during the first cycle; with increasing cycle number it decelerates. The activation process comes to a standstill at the 4^th^ to 5^th^ cycle (see Fig. 4). Because electrochemical activation of the surface is always linked with dilatation, tensions and cracks, *fresh* Si surfaces are continuously formed. As they are exposed to electrolyte they become immediately passivated, *i.e.*, they are subjected to SEI formation. Simultaneously, the Coulomb efficiency rises from 89% (1^st^ cycle) to 98.9% (5^th^ cycle) with an increasing tendency (Figs 3a and 4).

A conspicuous detail that can also be seen during the first half-cycle (see the black curve in Fig. 4) is the almost flat potential at 92 mV *vs* Li/Li^+^. It is not caused by any voltage limitation of the battery tester; instead, it rather represents an equilibrium potential of the anode during lithiation at a certain current density. Most likely, during the charging process the anode faces a lithium concentration gradient reaching from the surface to the subjacent layers that also get access to lithiation and, therefore, can store lithium. The Si regions located too far away from the surface remain non-lithiated; they act as both mechanical bearing and as current collector for the lithiated upper layers of the electrode (see below). During the subsequent cycles no such equilibrium potential seems to be established as the inner regions of the Si tower have already been activated, *i.e.*, they have already participated in the preceding lithiation process.

### Morphological changes

**Figure 5:**
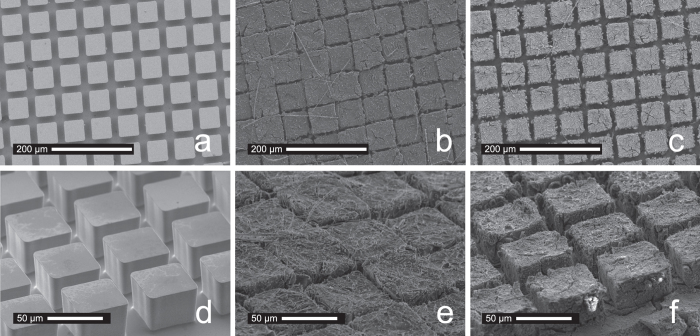
SEM images of the Si towers. High-angle view of (**a**) the native silicon towers, (**b**) the charged ones (5 cycles) and (**c**) the towers discharged again. (**d,e**) the same images but tilted by 60 degrees.

The monocrystalline silicon studied is, of course, subject to significant morphological changes during charging/discharging the battery (Fig. 5). In our case, scanning electron microscopy (SEM) served as a valuable tool to characterise the lateral dilatation during Li uptake. While the native anode reveals an even geometry with towers that are clearly separated by gaps with a width of 17.5 μm (see Fig. 5a), the charged one (5 cycles, 1105 mAhg^–1^) shows expanded towers being now in almost direct contact to each other (Fig. 5b). Discharging the anode does not re-establish the original tower-to-tower distance of the native anode (Fig. 5c). SEM images tilted by 60° show the differences in tower-to-tower distance between native, charged, and discharged states (Fig. 5d–f). The calculated lateral dilatation of the towers (50 × 50 μm^2^, see Fig. 5b), amounts to ca. 35%. Using the average tower diameters, as analyzed by SEM, the overall dilatation ranges from 31 to 37%. The distance between the towers (17.5 μm) and the extent of lithiation were optimized to each other in order that the configuration shows excellent reversibility in terms of Li insertion/extraction. With the lithiation level used here a larger distance between the towers would cause mechanical instability because the towers would not sustain each other.

**Figure 6:**
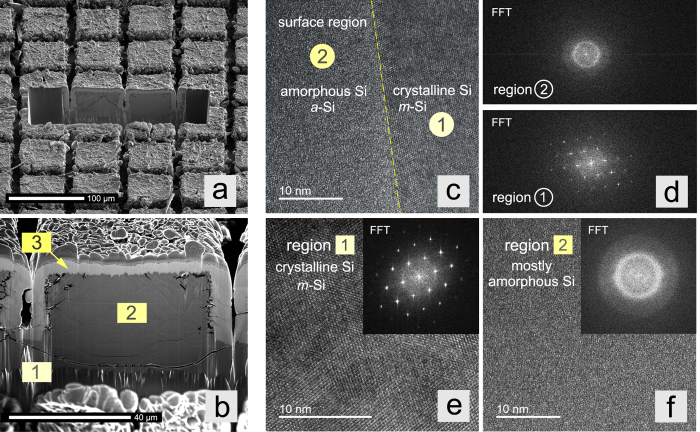
SEM image of a FIB-cut of discharged silicon towers after 5 cycles (Mos_1852); high-resolution TEM images used to differentiate between amorphous and crystalline regions of the lithiated Si towers. (**a**) SEM image, view on the towers, (**b**) SEM cross section view. (1) non-lithiated foot of the tower; (2) Si that took part in the lithiation/delithiation process and (3) capping layer of amorphous Si due to backscattering effects. See text for further explanation. The high-resolution TEM images in (**e**,**f**) prove that region (1) consists of crystalline Si, while region (2) (see 6b) is mainly composed of amorphous Si. Region (2), see Fig. 6b, does also contain some crystalline regions as is illustrated in (**c**,**d**); in (**c**) a high-resolution TEM micrograph near the surface region of a tower that has been lithiated and de-lithiated several times is shown. Insets in (**e**,**f**) show Fast Fourier Transforms (FFTs), in (**d**) the FFTs of the two adjacent Si phases are presented.

Cross sections using Focused-Ion-Beam (FIB) preparation techniques help make visible the morphology changes inside the anode material. In Fig. 6a two selected towers of the anode in the discharged state after 5 cycles are shown. The magnification in Fig. 6b reveals three different areas. A base layer of *m*-Si (1) that remained non-lithiated during the cycling procedures; it acts as current collector as well as mechanical bearing, as mentioned above. This layer exhibits no visual cracks. The major cross section of the tower (2, *a*-Li_*x*_Si and *m*-Si) acted as active material; it was subject to distinct volume changes. In contrast to the foot of the tower (1) cracks and structural displacements are visible. Worth mentioning, energy dispersive X-ray diffraction proved that during lithiation the large cavities inside the Si electrode were wetted with electrolyte. Area (3) reveals a bright capping layer of amorphous Si that was formed because of the effects of backsputtering; it simply represents a re-deposition artifact of the FIB technique^20^. Area (3) has been further investigated by bright-field high-resolution TEM, see Fig. 6e. For this purpose several lamellas were prepared and studied. In contrast to region (3), the upper part of the tower (region (2)) mainly consists of amorphous Si, see Fig. 6f. In both cases selected area diffraction supports this view. In the electrochemically active region, which is subjected to severe lithiation/de-lithiation (region (2)), residual Li-free crystalline regions pervade the mainly amorphous part of the towers. This observation is illustrated in Fig. 6c,d.

**Figure 7:**
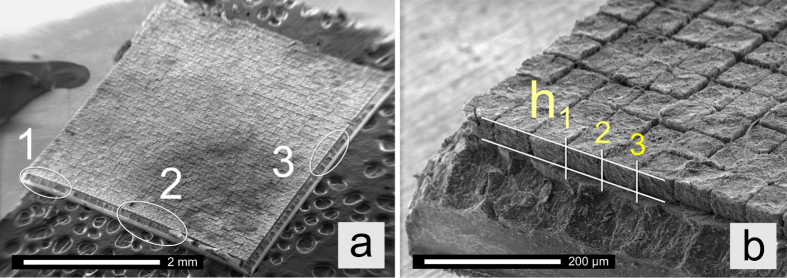
SEM image of a charged silicon anode after 5 cycles. **(a)** The areas marked served to determine the change in height of the towers after lithiation (a, Mos_1855). **(b)** Illustration of the principle to estimate the height of the towers (see area 1 in **(a)**).

In order to study the growth in height and the dilatation of the towers *during* lithiation, a charged anode (*Q* = 0.6 mAh) was transferred to the scanning electron microscope while permanently ensuring the protective inert gas atmosphere. As highlighted in Fig. 7 via the elliptical marks, three areas (1 to 3) of the anode electrode were investigated. Through measuring the changes in lengths seen via SEM taking into account the image tilt of the respective image (41° and 60°) we could estimate the differences in height of a native and a charged tower (see Table 1).

Volume expansion of the lithiated Si towers, considering all three measuring areas, is rather moderate. It is noticeable that the change in height of area 1 turned out to be the lowest. This finding can be explained by the exposed location of the corresponding towers. If situated at the corner of the rectangular electrode, the towers are susceptible to a higher contact pressure of the separator used. In general, this contact pressure redirects the expansion of the towers from a growth in height to a lateral increase. It plays a crucial role for the overall mechanical stabilization of the whole system resulting in highly promising cycleability at excellent specific capacities.

## Conclusions and Future Aspects

μm-sized Si towers prepared through semiconductor processing techniques turned out to be highly suitable for mass production of miniaturised rechargeable lithium-based batteries that can be directly used as power sources in integrated circuits. We used a μm-sized tower-shaped 3D patterned electrode whose towers are crystallographically oriented such that Li can easily break up the Si structure from the most preferred crystal orientation. Roughly speaking our cyclic voltammetry results on single crystalline Si are in line with earlier results obtained for polycrystalline silicon. In addition to voltammograms presented in the literature, the CVs recorded reveal at least three discrete reduction peaks during forming the amorphous Li_*x*_Si-phase. This observation suggests the presence of several X-ray-amorphous but short-range ordered cluster types in *a*-Li_*x*_Si. Cycling experiments on single crystalline Si electrodes show stable capacities with values higher than 1000 mAhg^–1^ at Coulomb efficiencies as high as 98.9%. SEM images of discharged Si towers do not reveal severe changes in shape. The patterned 3D structure consisting of the regularly arranged towers is clearly preserved after cycling. The whole system is able to reversibly endure lateral dilatations of up to 37% and perpendicular dilatations of up to 31%. A FIB-prepared cross section of cycled towers explain the spatiotemporal lithiation mechanism. Lithiation, being tantamount to amorphisation, starts at the electrode-electrolyte surface whereupon the *a*-Li_*x*_Si|Si interface moves deeper into the material with on-going lithiation. In the battery system presented here, single crystalline Si represents a bifunctional material: while the layers oriented toward the surface act as active material, those situated deep down fill the role of the current collector.

The excellent cycling performance at high energy densities combined with, in respect of Si, only moderate dilatation and morphology changes emphasises *monocrystalline* silicon as a highly practicable anode material. The patterned electrode, whose tower structure is scaled such that it optimally withstands volume expansion, is expected to perform even several hundreds of cycles in full cell configuration as preliminary tests have shown. Considering micro-applications, such as sensors, an extraordinary high cycle number is, however, not always required. As the energy density is rather high, in many cases the lifetime of the battery, if charged and discharged just a few times, would exceed that of the whole sensor.

## Methods and Experimental

### Preparation of the Si electrodes

The Si electrodes were prepared by deep reactive-ion etching using a highly boron-doped 8-inch silicon-wafer (monocrystalline, 200 μm in thickness, 〈100〉 orientation. The details of the anisotropic plasma etching of the wafer to yield laterally defined recess structures by means of an etching mask is described elsewhere^21^. The specific resistivity of the wafer-grade Si was 8 mΩ cm. The Si side of the electrodes was treated for 5 min with 5% (v/v) hydrofluoric acid and rinsed with deionized water to increase the electrochemical activity of Si by removing any SiO_2_ layers (see above). To eliminate any effects of moisture, electrodes were dried following a two-step procedure: at first, they were dried at 60 °C for 12 h, then in a second they were dried in high vacuum (<10^−4 ^mbar) for another 12 h.

### Electrochemical testing

Cyclic voltammetry and cycling experiments were performed using Swagelok based cells in three-electrode configuration with one 4 × 4 mm^2^ patterned Si sample that served as working electrode on a copper foil disc carrier with 12 mm in diameter. Li metal was used as counter electrode (CE); the reference electrode was also metallic Li. One layer of Whatman GF/B borosilicate glass fleece separator was wetted with an electrolyte of propylene carbonate (Sigma-Aldrich, anhydrous), 1 M LiClO_4_ (Sigma-Aldrich, battery grade). All cell components in contact with the electrolyte were dried for 24 h at 60 °C in fine vacuum, the Swagelok cells were assembled in a MB 150-B-G-II glove box (M. Braun GmbH, Garching, Germany) filled with dry argon excluding water, oxygen and nitrogen. Cyclic voltammetry experiments were carried out with a Biologic MPG2 (Bio-Logic Science Instruments SAS, Claix, France) at potentials ranging from 1000 mV to 100, 70 or 5 mV at a scan rate of 10 μVs^–1^; potentials refer to Li/Li^+^, *i.e.*, to the open circuit voltage potential of the anode during the initial cycle. Experiments consisting of 5 cycles were performed on a Maccor 4000 series battery tester (Maccor Inc., Tulsa, USA). For one cycle, the silicon anodes were charged (lithiated) in half-cell configuration using a current of 45 μA, starting at a potential of *E* = 1000 mV downwards until a charge quantity of *Q* = 0.6 mAh was reached; *Q* is equivalent to the charge capacity. After a resting step for one hour, the anodes were discharged at constant current *I* = 45 μA until a cut-off potential of 1000 mV was reached. This half cycle was completed after a rest time of 1 h. The same charging/discharging procedure was used to test the patterned anode in full cell configuration with Li(Ni_0.80_Co_0.15_Al_0.05_)O_2_ as cathode material. To calculate the specific capacity we determined the depth of the lithiated region (ca. 32 μm) by means of the FIB/SEM investigations shown in Fig. 6b. The volume of a single lithiated tower is 50 × 50 × 32 μm^3^. There are more than 3000 towers per cell resulting in ca. 5.4 × 10^−4 ^g Si serving as active material in one half cell; the density of Si is given by 2.336 g cm^−3^. With an absolute discharge capacity of 0.59 mAh this results in specific charge capacities as high as 1039 mAhg^−1^. Labels such as ‘Mos_1852’ represent the internal notation used to identify the cells tested.

### Scanning and transmission electron microscopy

SEM images were taken using a Zeiss Gemini DSM 982 (Leo Elektronenmikroskopie GmbH, Oberkochen, Germany) with a beam current of 0.1 nA and an accelerating voltage of 5 kV. The secondary electron images were recorded with an Everhart-Thornley detector with dwell times of 100 ms, a resolution of 1280 × 1024 and a tilt of 41°. The high-resolution TEM investigations were carried out with a Tecnai TF20 (FEI Company) equipped with a field emission gun featuring an acceleration voltage of up tp 200 kV. The microscope is equipped with a high resolution Gatan imaging filter including an UltraScan charge-coupled device camera (2048 pixels × 2048 pixels) that was used to capture the micrographs. The images were acquired in bright field TEM mode with an energy selecting slit (ΔE = 15 eV) to select only the elastically scattered electrons.

## Additional Information

**How to cite this article**: Sternad, M. *et al*. The microstructure matters: breaking down the barriers with single crystalline silicon as negative electrode in Li-ion batteries. *Sci. Rep.* **6**, 31712; doi: 10.1038/srep31712 (2016).

## Figures and Tables

**Table 1:** Change in height h_i = 1, 2, 3_ caused by lithiation of the Si towers; values refer to the initial height of the tower of 32 μm.

	area 1	area 2	area 3
tower 1, height (h_1_)	109.6%	133.0%	122.4%
tower 2, height (h_2_)	111.1%	129.8%	113.6%
tower 3, height (h_3_)	111.1%	—	—
**average dilatation**	**10.6%**	**31.4%**	**19.0%**
